# Retrieving Leaf Area Index (LAI) Using Remote Sensing: Theories, Methods and Sensors

**DOI:** 10.3390/s90402719

**Published:** 2009-04-17

**Authors:** Guang Zheng, L. Monika Moskal

**Affiliations:** Remote Sensing and Geospatial Analysis Laboratory and Precision Forestry Cooperative, College of Forest Resources, University of Washington, Box 352100, Seattle, Washington, USA 98195-2100; E-Mail: guangz@u.washington.edu (G.Z.)

**Keywords:** Leaf area index (LAI), remote sensing, light detection and ranging (LiDAR), gap fraction, gap size, terrestrial LiDAR

## Abstract

The ability to accurately and rapidly acquire leaf area index (LAI) is an indispensable component of process-based ecological research facilitating the understanding of gas-vegetation exchange phenomenon at an array of spatial scales from the leaf to the landscape. However, LAI is difficult to directly acquire for large spatial extents due to its time consuming and work intensive nature. Such efforts have been significantly improved by the emergence of optical and active remote sensing techniques. This paper reviews the definitions and theories of LAI measurement with respect to direct and indirect methods. Then, the methodologies for LAI retrieval with regard to the characteristics of a range of remotely sensed datasets are discussed. Remote sensing indirect methods are subdivided into two categories of passive and active remote sensing, which are further categorized as terrestrial, aerial and satellite-born platforms. Due to a wide variety in spatial resolution of remotely sensed data and the requirements of ecological modeling, the scaling issue of LAI is discussed and special consideration is given to extrapolation of measurement to landscape and regional levels.

## 1. Introduction

An important vegetation biophysical parameter, the leaf area index (LAI), is a dimensionless variable and a ratio of leaf area to per unit ground surface area. This ratio can be related to gas-vegetation exchange processes such as photosynthesis [1], evaporation and transpiration [2–4], rainfall interception [5], and carbon flux [6–8]. Long-term monitoring of LAI can provide an understanding of dynamic changes in productivity and climate impacts on forest ecosystems. Furthermore, LAI can serve as an indicator of stress in forests, thus, it can be used to examine relationships between environmental stress factors and forest insect damage [9]. Emerging remote sensing platforms and techniques can complement existing ground-based measurement of LAI. Spatially explicit measurements of LAI extracted from remotely sensed data are an indispensible component necessary for modeling and simulation of ecological variables and processes [10,11]. Since LAI remains consistent while the spatial resolution changes, estimating LAI from remote sensing allows for a meaningful biophysical parameter, and a convenient and ecologically-relevant variable for multi-scale multi-temporal research that ranges from leaf, to landscape, to regional scales [12].

The physiological and structural characteristics of leaves determine their typically low visible light reflectance except in green light. Past the visible, high near-infrared reflectance of vegetation allows optical remote sensing to capture detailed information about the live, photosynthetically active forest canopy structure, and thus begin to understand the mass exchange between the atmosphere and the forest ecosystem. Algorithms and models used as an input parameter to predict or estimate ecological variables have been developed using remotely sensed datasets based LAI [13–16]. For example, LAI obtained from optical remotely sensed data serves as a key parameter to estimate aboveground biomass of forest stands [17]. Due to recent availability, fine resolution spatial and spectral (hyperspectral) remotely sensed data are being used to retrieve LAI and other biochemical contents such as chlorophyll in leaves of forests [18–20]. Also in recent years, due to the emergence of light detection and ranging (LiDAR) techniques and equipment, numerous methodologies are being developed for point cloud datasets obtained from LiDAR to assess vegetation and forest three-dimensional structures [21–26]. The explicit three-dimensional information contained in LiDAR point clouds offers the ability to investigate forest health [27,28], forest stand structure and biophysical parameters [29–33]. Particularly, terrestrial LiDAR, with very high density point clouds, allows for improved retrieval of forest stand structure information including LAI [34,35]. Meanwhile, factors influencing the accuracy of leaf area density estimation have been investigated [31,36] including attention to leaf-on and leaf-off conditions [37, 38]. LiDAR has been used to monitor forest stands and environmental changes through the use of LAI as a key indicator parameter [39]. Currentely, due to single spectral band information deficiency, LiDAR has been combined with other hyperspectral remotely sensed datasets to obtain more comprehensive information about biophysical characteristics of forest ecosystems [40]. In recent years, a theory based on the spectral invariant property of leaves[41] has been applied to retrieve LAI and physical canopy height from optical sensors including single- [42,43] and multiple-angles [44]. The radiation budget theory characterizes the structural and spectral contribution in simulating the bidirectional reflectance factor in an efficient way and introduces new principles of photon-vegetation reflectance interaction, whereby one can characterize gap probability and gap fraction in terms of photon recollection probability and escape probability.

During past decades, efforts focused on LAI measurement strategy and theory, not only with ground-based field measurements, but also the retrieval of LAI based on array of remote sensors. In summary, there are two broad types of methods for estimation of LAI, either employing the “direct” measures involving destructive sampling, litter fall collection, or point quadrat sampling “indirect” methods involving optical instruments and radiative transfer models. The dynamic, rapid and large spatial coverage advantages of remote sensing techniques, which overcome the labor-intensive and time-consuming defect of direct ground-based filed measurement, allow remotely sensed imagery to successfully estimate biophysical and structural information of forest ecosystems.

A range of LAI definitions exist in the research literature, which complicates the comparison between works, and thus, the first focus of this paper is a compilation of LAI definitions. The second focus of the paper is the explanation of the gap fraction method theory. Thirdly, LAI estimation methods and sensors are discussed. Finally, remotely sensed LAI estimation and scaling issues associated with it are discussed.

## 2. Theory

In the early period of LAI research, due to the complicated distribution of foliage elements within the canopy, a modified Beer’s law light extinction model was developed. The model estimates LAI by mathematically analyzing light intercepting effect of leaves with different angular distribution based on a very common simplified assumption that all of foliage element and live parts within canopy are randomly distributed. The point quadrat method [45,46] was an early method used to mathematically analyze the relationship between projection area and foliage elements with all possible angular and azimuthal distributions. In this model, the extinction coefficient served as an important parameter to characterize the effect of leaves’ angular and spatial distributions on radiation interception. An algorithm was developed [47] to calculate extinction coefficients based on the assumption that the angular distribution of leaf area in a canopy is similar to the distribution of area on the surface of prolate and oblate spheroids. Because of the assumption of randomly located foliage elements within canopy, the LAI obtained from gap fraction [48] theory was not the true LAI, thus, a term called effective LAI was created to more accurately describe the result. However, gap fraction theory only applies to the percentage or proportion of gaps accounting for the whole hemispherical bottom-up view of a canopy. Gap size (dimensional information) is another very useful information to characterize clumping and overlapping effect, therefore, the gap size theory is a another stage for LAI ground-based filed indirect measurement development.

Recently LAI research focus has shifted from an empirical and statistical stage to process-based modeling stage due to the involvement of remotely sensed datasets and numerical ecological model implementation. The canopy structure in this paper is defined as the amount and spatial organization of aboveground plant materials including leaves, stems, branches, flowers and fruit, which affects the environmental factors such as air temperature, leaf temperature, atmospheric moisture, soil evaporation below the canopy, soil heat storage, leaf wetness duration and others [48]. The physical dimension (size, shape), relative position, spatial arrangements between different canopy elements determine the amount and spatial distribution of fraction of photosynthetic radiation (fPAR) within and below the canopy, which control the absorption, reflectance, transmission, and scattering of solar radiation. A single live leaf reflects green light and near-infrared light due to its internal structure. When scalling to the individual tree or forest stand level, non-random distribution and multi-layer structure of canopy elements result in multiple scattering of radiation between the different layers of foliage elements and other parts of canopy. This results in the obvious difference in reflectance for the individual leaf, tree canopy and a stand at landscape level. The denser a canopy, the more absorption and reflectance of solar radiation occurs and less energy is transmitted to the ground surface below. In addition, the difference of reflectance properties at various scales is dependent on the field of view (FOV) and spatial resolution for various sensors. The shadows between tree canopy and hot spot result from the relative position of sensor and sun (the hotspot is a phenomenon that occurs when the sensor sees only sunlit elements). Geometrical optical (GO) models such as bidirectional radiative directional function (BRDF) [49] and 5-SCALE radiative transfer model (RTM) [50] were developed to simulate the reflectance properties at different scales. Clearly, vertical and horizontal canopy structures are becoming an indispensible input parameter for modeling ecological process such as photosynthesis, evaporation, transpiration and carbon sequestration of forest ecosystems. In terms of the economic value of a tree, the tree bole is linked to tree stem volume, timber production, and the characterization of forest inventory. From the ecological perspective, foliage is applied in modeling biological processes at leaf-level and the foliage distribution is a key factor affecting competition for resources, such as light, nutrients and moisture of intra- and interspecies of forest community stand. The most important canopy attributes affecting solar radiation penetration through canopy and indirect LAI measurements are leaf angular distribution and leaf spatial distribution. On one hand, leaf angular distribution affects radiation transmission through canopy at different angles; on the other hand, leaf spatial distribution affects the amount of radiation transmitted through the canopy.

### 2.1. Definitions

During past decades, definitions of LAI have been provided by scientists from many disciplines for a range of purposes, such as determination of forest community succession, simulation of potential biological activities, and solar radiation regimes within plant canopies. The definition of LAI are summarized and compared in Table 1. A common and acceptable definition of LAI needs to be addressed to make research results comparable.

As shown in Table 1, total leaf area index (ToLAI) was first defined as the total one-sided area of photosynthetic tissue per unit ground surface area [51,52]. This definition is especially applicable to flat broad leaf condition with same area on both sides of leaf. In reality, the shape of leaves is not always of this type [53], some leaves such as white spruce (*Picea glauca*) are needle-shaped and arrangement is spiraled. Each needle has an approximate cylindrical shape which this definition cannot describe accurately, thus, projected area of leaves has been provided [54]. Projected LAI (PLAI) is defined as the horizontal area that is cast beneath a horizontal leaf from a light at infinite distance above it. The cumulative LAI (unitless) of a canopy by calculating the sum of vertical projection of foliage area on a horizontal plane from ground (z = 0) to top of canopy (z = h) [55]. LAI depends on the average surface density coefficient of the foliage (u) expressed in m^2^/m^2^:

(1) $$L(z)=\int_{z=0}^{h}u(z)\text{dz}$$

The concept of silhouette leaf area index (SLAI) was introduced and defined as the area of leaves inclined to the horizontal surface, and was compared with TLAI and PLAI to investigate the effect of leaf orientation on radiation interception, it was shown that the leaf orientation effects, or shading, or both, caused more variation in the interception of solar radiation than did variation in leaf geometry [56]. Effective leaf area index (ELAI) was defined as one half of the total area of light intercepted by leaves per unit horizontal ground surface area based on the assumption that foliage elements randomly distributed in space, and was introduced to precisely describe the shortwave and long wave irradiance condition under a Douglas fir forest stand [57]. Trees usually have differently-shaped canopies and foliage elements which means that a general definition of LAI needs to be obtained. The most popular and widely accepted definition of the true leaf area index (TLAI) is defined as one half of total leaf area per unit surface ground area [58,59] by mathematically analyzing mean projection coefficient for various perfect geometrical objects representing the different shapes of real leaves.

### 2.2. Canopy Distribution and Leaf Inclination

Based on the assumption that the forest canopy is randomly-distributed, the solar radiation regime was simulated to obtain the amount of penetrated beam radiation through the canopy structure and develop the algorithms to estimate the LAI. According to Beer’s Law [60], when a beam of monochromatic radiation passes through a compound, absorbance and transmittance takes place and the radiation will be attenuated. Likewise, when a beam of solar radiation transmits through the canopy, the leaves will absorb some of the visible light and reflect some infrared light which results in the changes between the solar radiation before and after passing through leaves. The extinction coefficient [47] was developed to describe the canopy function when shifting the beam radiation.

**Figure 1.**
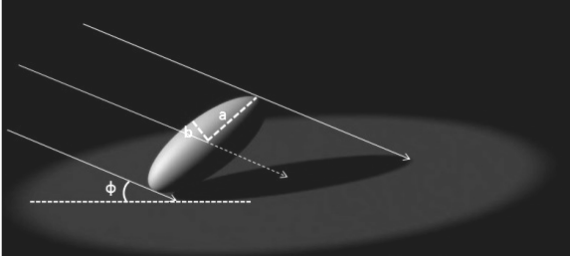
Schematic diagram to illustrate the shadow area calculation for ellipsoid (created based on Campbell [46]).

The extinction coefficient represents the area of shadow cast on a horizontal surface by the canopy divided by the area of leaves in the canopy or the average projection of leaves onto a horizontal surface [47]. Among the many geometrical objects, the sphere, cylinder, and cone models provided simple methods to calculate the extinction coefficient, the Figure 1 shows an example of extinction coefficient calculation of ellipsoid, and the following equations calculates the shadow area of ellipsoid under parallel light source:

(2) $$A_{s}={\pi b}^{2}{\left[1+a^{2}/(b^{2}\;{\text{tan}}^{2}\;\varphi )\right]}^{½}$$

where A_s_ is the shadow area of ellipsoid under the illumination of parallel light source, a and b are the long and short axis of ellipsoid respectively, ϕ is the inclination angle of direct solar beam. The density functions for foliage inclination angle are generally crude approximations to actual foliage inclination angle densities. Thus, the probate and oblate spheroids were proposed to approximate the actual foliage spatial distribution and a more flexible and general equation was developed to calculate a more accurate extinction coefficient of a forest canopy [47]. Based on the ellipsoid model, a leaf angle density function for canopies has been provided and the leaf inclination angle density function is a fundamental property of plant canopy structure and is needed for computing distribution of leaf irradiance [61].

### 2.3. Gap Fraction

**Figure 2.**
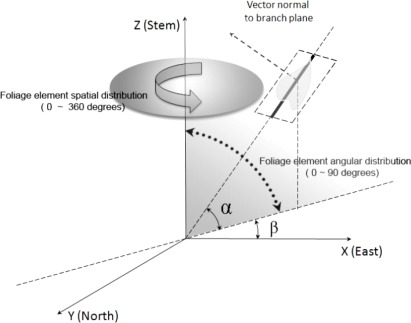
Schematic diagram illustrating the spatial and angular distribution for a single leaf (β is the azimuthal angle for this individual leaf ranging from 0 to 360 degrees, and α is the inclination angle of this single leaf, whose range is from 0 to 90 degrees).

Among the indirect methods to estimate LAI, one popular way is to measure light penetration and the amount and distribution of openings in the canopy which is often referred to as gap fraction [48,62]. Gap fraction describes the possibility of sun light rays not penetrating into the understory through the canopy. The spatial position of each single leaf in reality is determined by its spatial and angular distribution, which is shown in the Figure 2.

The measurement of gap fraction is generally an acceptable way to analyze the structure of a tree canopy and often parameterized with the LAI and leaf angle distribution. P (θ), which denotes the gap fraction at the zenith angle θ of incoming direct sun light, which can be expressed mathematically as the Poisson model:

(3) P(θ)=exp[−G(θ)×L/ cos (θ)]

where G(θ) is the projection coefficient of the foliage onto a plane (normal) perpendicular to incoming radiation [63,48], and L is the LAI of the forest canopy including all aboveground structural components (branches, boles, cones, and epiphytes).

(4) G (θ) / cos (θ)=K

K represents the average projected area of the canopy components on a horizontal plane. It is assumed to link to the extinction coefficient discussed above.

**Figure 3.**
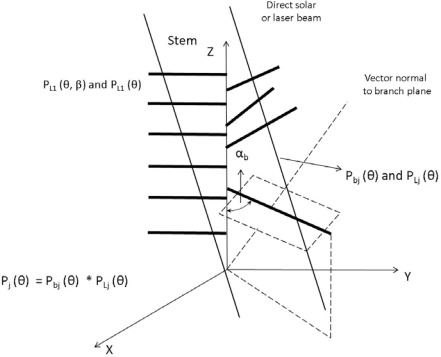
Schematic diagram illustrating the multi-layer theoretical model to calculate the gap fraction.

Due to the multilayered structure of a forest canopy, many gaps form within the canopy and allow solar beams to penetrate through and provide enough light for understory growth (Figure 3). This method is based on an assumption that the spatial distribution of foliage is random; the overlapping and clumping of leaves within the canopy has not been considered, thus, the LAI obtained in this way is not the true LAI, but underestimated LAI. Based on the Miller theory [64] and Chen definition [57], the definition of effective LAI is:

(5) $$L_{e}=2\int_{0}^{\frac{\pi }{2}}\text{ln}[\frac{1}{P(\theta )}]\;\text{cos}\theta \;\text{sin}\theta \;d\theta$$

where *P*(θ) is the measured canopy gap fraction at zenith angle θ and L_e_ is the effective LAI.

Chen also pointed out that an important consideration implicitly expressed in (4) is that LAI can be calculated without knowledge of foliage angle distribution if the gap fraction is measured at several zenith angles covering the full range from 0 to π/2 [65].

Figure 3 shows the theoretical model used to calculated gap fraction for multi-layer canopy structure forest stand. The canopy is divided into two levels: branch level and leaf level. For each layer, each branch is composed by a sub-branch with attached leaves and perforations. The probability of penetrating the perforation between leaves P_Lj_(θ) and branched P_bj_(θ) in j^th^ layer are calculated respectively, thus, the probability of solar beam penetrate the j^th^ layer is the product of probability of penetrating leaves and branches respectively P_j_(θ) = P_bj_(θ) × P_Lj_(θ), P_L1_(θ, β) and P_L1_(θ) represent the probability of direct solar beam penetrate the leaves in first layer of canopy where the incident angle of solar beam is θ and azimuthal angle is β.

Besides the theoretical formula and analytical expression described in Figure 3, an improved algorithm has been developed by Nilson to estimate the canopy indices and LAI from gap fraction data [66], the method used the eigenvectors and eigenvalue of the covariance matrix to describe the random variation of gap fraction at the near-zenith view direction and showed good performance in relatively open boreal and sub-boreal forest environments.

Gap fraction is usually obtained automatically using optical radiation measurement instrument such as hemispherical photograph, or LAI-2000 (Li-Cor, Inc). A key component of this method is to set up the optimal threshold to separate the leaves from sky. Usually overexposure will result in an overestimated projected LAI and underexposure will make the projected LAI much higher. Different digital hemispherical photographs which were collected under a range of sky brightness conditions for an array of forest species and openness have been compared [67]. Zhang [67] found that the automatic exposure is apt to underestimate the effective LAI and provides a protocol for taking the digital hemispherical photography in different open-canopy conditions.

Many commercial optical instruments based on the gap fraction theory are available to estimate the effective LAI. All of the instruments can be divided into two broad types including linear sensors such as DEMON, line quantum sensors, and the other type are hemispherical sensors such as LAI-2000 (Li-Cor, Inc), the leaf laser, hemispherical photography and the CI-100. Unfortunately, these usually underestimate the LAI of forest trees due the assumption of random distribution of foliage.

#### 2.3.1. Clumping and Gap Size

There are two causes that affect the accuracy of LAI estimation. The first is the non randomly-distribution of tree foliage resulting in overlapping and clumping between the leaves within canopies. If we want to obtain true LAI, these effects should be carefully considered and incorporated into the LAI estimate. The other cause is light obstruction from canopy components such as branches, boles and stems, especially for conifers on which needles with a shoot will be significantly clumped.

The fraction characteristic of sunfleck for obtaining effective LAI has been well studied under the “gap fraction theory”. Because the sunflecks’ size and their spatial distribution under canopy result from the gaps in the non-randomly distributed overlaying canopy in the Sun’s direction, the structural characteristics of the sunfleck are an important information source. If the quantitative correlation between sunflecks’ distribution and frequency and foliage clumping and overlapping effect can be identified, such information is sufficient to translate effective LAI to true LAI based on this relationship denoted by gap size theory. This procedure is summarized below.

In order to quantitatively describe the sunfleck dimensional information, a theoretical model focused on the size and shape of sunflecks under forest canopy needs to be developed. The model uses the sunlit segments along a straight-line transect under the forest canopy to represent the sunfleck size distribution [68], and the probability distribution of shadow-edge angles information (penumbral effects of the finite solar disc) to predict the shape of sunflecks [69]. By combining the gap-size theory and penumbral effect, the light intensity under the plant canopy can be predicted quantitatively and used to accurately and spatially estimate moisture evaporation and photosynthesis of leaves. The sunfleck distribution, direct solar radiation and diffuse skylight are related to the geometrical structure of plant stand, thus Nilson [70] proposed a theoretical model to analyze the gap frequency of forest plant stands based on Possion, positive and negative binomial distributions. The Markov processes theory was also presented by Nilson [70] to predict the gap frequency for stand geometry. Nilson [70] recommended that the binomial and Markov model be used for practical use side by side in order to avoid the unrealizable Poisson model. All three models are based on the assumption of randomly distribution of foliage elements.

Two different gap-size theories were developed by Chen and Black [71] (hereinafter referred to as theory one) and Chen and Cihlar [72] (hereinafter referred to as theory two) to evaluate the effect of foliage clumping at scales larger than the shoot, and the term “clumping index” was given for this effect. The clumping index can be measured by using the sunfleck-LAI instrument Tracing Radiation and Architecture of Canopies (TRAC, 3rd Wave Engineering) [73]. The major difference between these two methods is the dependence on randomly spatially distributed foliage element. Theory one developed a Poisson model to describe sunfleck size distribution under clumped plant canopies based on the assumption that foliage clumps are randomly distributed in space and foliage elements are randomly distributed within each clump. Although it improves the result of LAI estimation without considering the canopy architectural information, it is still not reliable due to this assumption. Theory two developed a gap-size measurement model which can be used for any heterogeneous canopies and is the theoretical foundation of prototype sunfleck-LAI measurement instrument TRAC. It’s an improvement on the finite-transect method because it avoids the assumption of local randomness. Thus, theory two avoids making the assumption for a spatial distribution pattern of foliage clumps used in the theory one and is applicable to various of plant canopies.

The Poisson model was first modified by considering the non-random spatial distribution of canopy elements and expressed as [63]:

(6) P (θ)=exp [− G (θ)×Ω×L/ cos (θ)]

where G (θ) characterizes the leaf angle distribution. Ω is a parameter determined by the spatial distribution pattern of leaves. When the foliage spatial distribution is random, Ω is 1. If leaves are regularly-distributed (extreme case: leaves are laid side by side), Ω is larger than 1. When leaves are clumped (extreme case: leaves are stacked on top of each other), Ω is less than 1. Foliage in plant canopies is generally clumped, and hence Ω is often referred to as the clumping index [74]. L is the LAI of a forest canopy including all aboveground structural components (branches, boles, cones, and epiphytes). In terms of conifer trees, the clumping index Ω can be separated into two parts:

(7) $$\Omega =\Omega_{E}/\gamma_{E}$$

where Ω_E_ is the stand-level clumping factor at scales larger than shoot and γ_E_ is the clumping at shoot level and was named after “needle-to-shoot area ratio” [74]. As for deciduous trees, γ_E_ is equal to 1.

In order to quantify the clumping effects, Chen thought that there are two underlying assumptions of this correction method: shoots are the basic unit responsible for light interception, and shoots randomly distribute within a canopy [72]. In addition, the research found that the non-randomness of shoot position reduces indirect measurement of LAI by approximately 35% for a Douglas-fir canopy. In this situation, there are three components that constitute the percentage of measurement of LAI. Non-randomness of shoot position accounts for 35%, the indirect measurement through destructive sampling captures 31%; the remaining 34% can be explained by the clumping of needles with shoots. The effect of needle clumping with shoots can be obtained by measuring the ratio of half the total needle area in a shoot to the shoot intercepting area.

By combining gap fraction and gap size theory, true LAI can be obtained for individual trees. The regional, landscape, or even global LAI spatial distribution or variation can be acquired using an airborne, or satellite platform based sensors, along with the specific algorithms applicable to the characteristics datasets collected by these platforms.

## 3. Measurement Methods and Sensors

Among the range of methods used to estimate LAI, there are two broad types: direct and indirect.

### 3.1. Direct Methods

In terms of the direct method, leaf collection and leaf area determination techniques are often used. The leaf collection can also be implemented by harvesting method through destructive sampling which collects and removes green leaves from a sample plot or some representative trees from a plot, or by non-harvesting litter traps through collecting the leaf litter during the autumn leaf-fall period for deciduous trees [75]. The planimetric and gravimetric are two different kinds of methods of leaf area determination techniques in terms of direct method. Planimetric approach is based on the correlation between the individual leaf area and the number of area units covered by that leaf in a horizontal plane; and the gravimetric method was implemented according to the correlation between dry weight of leaves and leaf area using predetermined leaf mass per area, once the leaf mass per area is known, the whole sample is dried to calculate the leaf area from its dry-weight and sub sample leaf mass per area [75].

### 3.2. Indirect Methods

The indirect point quadrat and allometric constitute the contact method. The inclined point quadrat method was first elaborated in 1930’s [76,77], and further developed by Warren Wilson [78,46], the theoretical study in this work revealed that 57.5 degrees of point quadrat inclination angle is the best one to avoid the variation in relative frequency resulting from differences in foliage angle. The relative frequency is recorded by point quadrats measures is the area projected in the direction in which the quadrat lies. But this result was questioned when it was applied to plant canopy with branch architecture (with needle-clumped shoot), it should be adjusted to 62 degrees in order to estimate the effective LAI, and was tested in the Douglas-fir forest stand [79]. In this work, the Poisson model was used to describe the geometry of tree branches (thin slabs of foliage in which leaves are confined), based on the assumption of randomly dispersed leaves in a single layer of canopy, the values of effective LAI obtained from this model were approximately 55–65% of the true LAI.

The allometric method is based on the relationship between leaf area and other parts of the woody plant elements that support the green live leaf biomass (such as stem, branch diameter, woody to total area fraction, tree height). However, this relationship is not reliable due to the seasonal change, forest health condition, local climate condition, and stand density. It’s also a species or site specific statistical relation [75].

In addition, the non-contact indirect method is the most popular and convenient way to estimate LAI in practice. According to the working way of instrument or sensors, passive and active are two common categories for retrieving LAI in different spatial scales ranges from leaf, forest stand, landscape, to region or even global levels.

#### 3.2.1. Passive Sensors

##### Terrestrial

Without contact with leaves, based on the radiation transmission and gap fraction theory discussed in Sections 2.2 and 2.3, an array of commercial optical instruments has been developed to estimate effective LAI such as Plant Canopy Analyzer (PCA) (LI-COR, Lincoln, NE), DEMON (CISRO, Center for Environmental Mechanics, Canberra, Australia), Ceptometer (Decagon Device, Pullman, WA) and digital camera with fisheye lens. Other instruments characterize the clumping and overlapping effect within plant canopy with branch architecture based on the gap size theory described in section 2.4, this includes the TRAC instrument (3^rd^ Wave Engineering, Ontario, Canada). A certain weather condition are required to implement different instruments, for example, cloudy sky is the ideal condition for PCA and digital camera with fisheye lens, which are used to capture the “gaps” between foliage elements, however, the TRAC needs the clear sunny days to take the measurement because of the reference value of solar direct radiation is needed to quantitatively locate and discriminate the gaps and their dimensional information.

All airborne and satellite optical remote sensing, especially hyperspectral remote sensing [80], are aimed at retrieving the spectral characteristic of leaves, which are determined by the internal bio-chemical structure and chlorophyll content of leaves. This basic and important spectral characteristic can be measured by the spectroradiometer for individual leaf or forest canopy at the terrestrial scale. For example, the portable field spectroradiometer FieldSpec Pro FR (Analytical Spectral Devices, Inc. Boulder, USA) is designed to collect solar reflectance, radiance and irradiance measurements. High spectral resolution (1nm interval) with a 350 nm – 2,500 nm spectral range is ideal for vegetation mapping and monitoring applications. The high resolution spectrum measurements are important inputs of the leaf-level model (modified PROSPECT [81]) and geometric optical model (5-scale) [82] for retrieving leaf biochemical contents from hyperspectral remote sensing images.

##### Airborne

Compared with the satellite images, the aerial images from airborne remote sensors have much finer spatial resolution, however, because of this, the shadows resulted from the obscurance of tree canopies between each other bring biases into the LAI estimation from the airborne optical remote sensors. This complicates the simulation of the radiation regime from optical remotely sensed data without the use of a geometric optical model. Due to different angular distribution of foliage elements, and in forest canopies, the solar radiation interacts with the foliage at four different scales: within groups of trees, within individual crowns, within branches, and within shoots. In the geometric-optical model, according to the calculated shape of canopy crowns and spatial distribution of all canopy elements, the proportion of shadows cast as a function of view direction relative to the hot spot direction can be calculated and simulated, at the same time, the corresponding spectral characteristics can be obtained based on the geometrical shape and arrangement, the spectral reflectance of individual tree or whole canopy can be simulated by using the geometric optical model (5-scale) [82]. Therefore, it’s a basic and theoretical foundation for the multi-angular remote sensing throug observing the forest canopies from different view directions shadows between canopies can be effectively eliminated and increase the accuracy of monitoring of changes and patterns of forest stands at different scales. LAI extraction was demonstrated for various crop species in a given region based on high-resolution remote sensing data [83]. In this study, an inversion modeling approach was used on multi-temporal remote sensing to retrieve the LAI spatial seasonal variation distribution and created the look-up table of LAI based on the values modeled using the PROSPECT-SAIL radiation model by inputting *in situ* measurement data and literature values served as a reference tool. Other literature [84] reported that imaging spectrometer data was used to retrieve spatially explicit information on canopy structure and foliage water content in order to assess first risk and to manage the impact of forest fires. Two hybrid canopy reflectance models, GeoSAIL and FLIGHT, were used to simulate the canopy reflectance of the observed heterogeneous forest stand. The results demonstrate the feasibility of estimating structure and foliage water content of a coniferous canopy based on radiative transfer modeling, and was validated by ground field measurement. This method is more favorable than the traditional empirical method relying on the relationship between the LAI and vegetation index.

The airborne multi-spectral remote sensors were extensively used to retrieve LAI by correlating the spectral information from remotely sensed data and ground based measured LAI values [85]. For example, Airborne Imaging Spectrometer for Applications (AISA) radiometer was used to capture the crop response through monitoring and mapping the LAI variogram map using the residual maximum likelihood method, and the result derived from airborne images was cross-validated with the LAI estimation result from kriging interpolation method [86]. An eleven-band Daedalus AADS 1268 ATM airborne multi spectral sensor (MSS) was employed to acquire the spectral information for grass land in study area [87]. Since the availability of satellite images with finer resolution and more extensive coverage, most LAI studies are based on satellite- remote sensing data.

##### Satellite

The premise of retrieving LAI based on spectral remote sensing data relies on the the unique spectral response characteristic of green leaves compared with other land surface materials. The selective absorption of solar radiation of green leaves, the high absorption of visible light, and much more red light than infrared light make it possible to generate vegetation indices such as Simple Ratio (SR) [88], Normalized Difference Vegetation Index (NDVI) [89], Enhanced Vegetation Index (EVI) [90], and Reduced Simple Ratio (RSR)[91]. It shows that the RSR is better than NDVI or SR for estimating LAI since the RSR is more sensitive to the change of LAI [92], in addition, the RSR can be effectively improve the LAI retrieval in the boreal forest of Canada, the shortwave infrared (SWIR) signal used in RSR calculation reducing the background effects and increase sensitivity, and the land cover map may not be required prior to LAI mapping, a greater improvement can be made by introducing SWIR into RSR for r^2^ of LAI estimation in jack pine (30%) from 0.554 to 0.662 and black spruce stands (15%) from original 0.501 to 0.578[91]. EVI was developed as satellite vegetation product for the Terra and Aqua Moderate Resolution Imaging Spectroradiometers (MODIS), it can improved sensitivity in high biomass regions while minimizing soil and atmosphere influences by calculating the combination of red, near infrared and blue bands. But there are some limitation of EVI due to the blue band which make it difficult to generate long-term EVI time series product, thus, a two bands EVI has been developed [93].

Landsat series sensors (thematic mapper, TM)/(enhanced thematic mapper, ETM^+^) are commonly used due to their balance between spectral, spatial, and temporal resolutions. Based on the various regression relationships between the many vegetation indices and LAI, the linear and non-linear estimation model has been developed to estimate and map LAI at the landscape and global levels [94, 15]. In the boreal forests of Canada, r^2^ has achieved values between 0.38 and 0.66 [95]. In addition, regression and geostatistical methods were compared for mapping LAI using Landsat ETM^+^ datasets in boreal forest of Canada [13]. Due to the impracticality of obtaining reference LAI measurements, it is suggested that a non-spatial regression method (reduced major axis) should be used to simulate regional NPP. One major issue of retrieving LAI from vegetation index calculated from the different band combinations of multi- or hyper-spectral sensors is the saturation of LAI, which means the vegetation index and LAI will not increase linearly, and the complicated structure of forest canopy such as the angular distribution of foliage element and canopy structure will affect the reflected radiances. Furthermore, the effects of understory and soil back ground need to be considered. In addition, since the regression relationship are site-, time- and species specific, these cannot be easily applied in a landscape, or global level.

Hyperspectral remote sensing datasets have a fine spectral resolution allowing for the detection of physiological characteristics such as accurate chlorophyll a and b content estimation [96]. The relationship between hyperspectral vegetation indices and LAI has been examined for wheat and chickpea over their growth cycles [97]. Not only did the examination focus on the leaf level, but also the canopy structural variables inversely estimated based on a reflectance model from hyperspectral remote sensing data [98]. Moisture condition is an important indicator for vegetation stress [99] due to forest insects such as mountain pine beetle the water content can be easily detected and monitored by remote sensing data over large spatial and time scales, especially with hyperspectral remote sensing data. Due to the fine spectral resolution of hyperspectral remote sensing data images, the reflectance inverse model, along with the hyperspectral indices was used to estimate the leaf and canopy water content in poplar plantations [100,18]. LAI estimation based on hyperspectral data has been done both in croplands [101,102] or forest stands [40]. The numerous band information of hyperspectral remote sensing data will unavoidably bring much redundant information, thus a compression algorithm must be employed to be efficiency, thus, the significance of the of data compression on the retrieval of leaf chlorophyll content and LAI for precision agriculture application from hyperspectral data has been assessed [103]. In terms of prediction power and stability, broadband and hyperspectral vegetation indices are better for estimation of green LAI and canopy chlorophyll density [104].

Another important aspect of LAI estimation is the time series issue, and not only the spatial distribution of LAI in a given region with a single time. Much of ecological models are designed to estimate those with time-series changes of ecological variables such as Net Primary Productivity (NPP); therefore LAI as a key input parameter is also needed first to get the time-series spatial distribution based on remote sensing data. Sometimes due to non-availability of Landsat images for a certain time range, the monthly LAI variation images cannot be obtained. Some work has been done with the help of MODIS datasets to get the Landsat LAI time series imagery [105]. MODIS LAI [42, 43] product, which is an eight-day interval time series, images with 250-meter spatial resolution and has a total of 45 scenes for a whole year in this region. The variation trend curve can first be retrieved based on the MODIS-LAI product, and then applied to Landsat images to generate time-series Landsat images which will be inputted into a process-based model to simulate the whole year NPP variation and spatial distribution. A novel and simple approach to optimal interpolation analysis of LAI using MODIS data has been developed to make MODIS-LAI products more appropriate for environmental prediction than the original data [106].

#### 3.2.2. Active Sensors

Without receiving the reflected solar radiation by land surfaces, the active remote sensors emit a certain wavelength single and capture the echoes reflected by target objects. Radio Detection and Range (Radar) and previously defined LiDAR are two common active remote sensing systems. Radar emits electromagnetic waves (such as microwave or radio waves) to identify the range by capturing the waves reflected by the target and detected by a receiver. So it’s possible to detect the structure information and physical dimensional information for forest stands and individual trees, even the biophysical parameters such as LAI using Radar system. The responses of microwave Radar to LAI, canopy moisture, dry weight of wheat, corn and sorghum were investigated as early as 1980s [107]. LiDARis a relatively new remote sensing instrument used in forest application. Compared with the passively receiving spectral reflectance signals from land surface objects for optical remote sensing sensors, LiDAR systems actively emit a wavelength laser light (such as green or near infrared), the laser beam will transmit through its straight path until it is changed by the encountering object. Terrestrial, airborne and satellite LiDAR system can be sorted out according to the based platform. Also, discrete and fullwaveform are two common LiDAR systems. The discrete LiDAR system, provides single or multi- returns for each laser pulse LiDAR, the fullwaveform LiDAR provides a waveform for one pulse. For discrete LiDAR usually three or more echoes bounce back to the sensor for each laser pulse. Terrestrial LiDAR is a discreet pulse system but with only one return for each laser pulse. Discrete LiDAR systems measure the distance between sensor and objects by recording the time of flight of laser. Each laser pulse return records two different kinds of information including spatial position (x, y, z) coordinates, and an intensity value. Only two dimensional information provided by optical passive remote sensing, but active remote sensing has shown ability to capture more details about three-dimensional structure information. At the same time, it overcomes some of the disadvantages of passive remote sensing such as cloud-cover issues and vegetation index saturation problems. By using an active remote sensing system, one can subjectively choose a study area of interest.

##### Terrestrial

**Figure 4.**
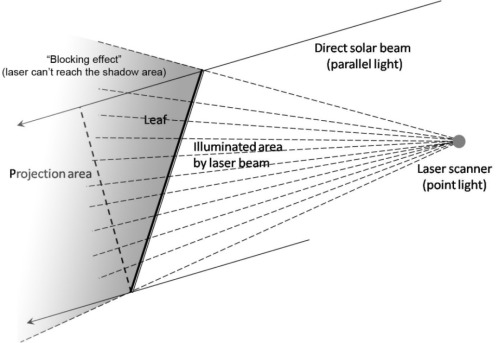
“Blocking effect” between direct solar beam and point light source laser scanning.

The non-random and complicated spatial and angular distribution of leaves within canopies requires one to obtain more details in order to retrieve the true LAI. In particular, terrestrial LiDAR makes it possible due to its high-density laser pulse returns, but there are still some issues that need to be carefully consider. The first question considers the influence of terrestrial LiDAR set-up on the accuracy of retrieving forest stand structural information [108], the laser scanning pattern will greatly affect the amount of information one will obtain due to the “shadow effect”, according to the different location where scanner sits, there are various geometrical scanning patterns including the lateral sideway scanning, bottom-up hemispherical scanning, top-down canopy crane scanning. The appropriate geometric scanning way should be chosen based on the specific study purpose, if one wants to capture the height of a tree, the top-down scanning should be taken, however, the bottom-up scanning way will be ideal to predict the gap fraction by analogy with the digital hemispherical photos. In the gap fraction theory, the extinction coefficient [47] is a very useful parameter to characterize the blocking effect of leaves with various shapes under parallel light source (i.e. direct solar beam), as for the laser generated by terrestrial LiDAR system, since the distance between the light source and object are close enough to consider it as a point light source (i.e. laser beam scanning), instead of parallel light source. Blocking effect occurs when the laser beam cannot reach some areas obscured by the first object it encounters in the transmission path, which is defined as a ratio of the area illuminate by laser beam over the total surface area of the object, the blocking effect describe the obscure of leaves with different shapes under the point light source. The most obvious difference between the laser beam and direct solar beam is the area of object illuminated by different light sources, in the case of the direct solar beam, it’s parallel light, but for the laser scanning, because the position of laser scanner is fixed, it should be treated as point light source, the difference of blocking effect between parallel and point light source is shown in Figure 4.

After getting the parameter of blocking effect, a 3-D gap fraction method should be developed for discontinuous canopy for different species from different view directions, by following the procedure described in Figure 1, the effective LAI can be acquired from the 3-D gap fraction method. Then, due to the fine resolution of laser beam (1mm can be achieved by Leica ScanStation 2, for example), the gap size theory applicable to 3-D point cloud data produced by terrestrial LiDAR needs to be developed to characterize the clumping effect and translate the effective LAI to true LAI.

A significant difference between terrestrial and airborne LiDAR are the limited ground laser pulse returns from terrestrial LiDAR systems due to its natural scanning angle and position. The terrestrial LiDAR ground point area also distributed in uneven frequencies, where more points tend to be collected near the scanner and fewer points farther away from the scanner; this issue is exasperated by the angel at which the ground faces the scanner. This means that the gap fraction and effective LAI cannot be obtained according to the ratio of ground laser pulse returns and total returns which has been widely used to characterize canopy coverage based on airborne LiDAR [109,110]. 3-D voxel model is a popular method to process the point cloud data generated from terrestrial LiDAR, and it is also used to calculate the plant area density [35]. There still numerous theoretical problem needed to be resolved regarding LAI retrieval from terrestrial LiDAR, for example, how to separate the photosynthetic and non-photosynthetic parts in the point cloud data for an individual tree or forest stand? In other words, the woody area is difficult to be obtained for evergreen trees. Since the point cloud data is discrete, translating it into a raster image is still an issue without losing accuracy. Because of the “block effect”, the accuracy assessment of final LAI result requires the understanding of the information loss between the point cloud data and true tree.

##### Airborne

Not much work has been done on LAI retrieval from radar sensors, some are trying to build the relationship between radar signals and LAI [107,111,112]. However, extensive work on the application of airborne LiDAR systems in forestry has been done [113–115]. Methodologies based on LiDAR datasets have been developed to assess three-dimensional forest structures [21–26]. Due to the advantage of sufficient three-dimensional information obtained from terrestrial and airborne LiDAR systems, these have been used to estimate defoliation during forest insect outbreak [27,28]. Morsdorf *et al.* [110] reported that estimation of gap fraction and LAI from small footprint airborne LiDAR, the first echo of laser pulse capture 73% variation of fractional cover with a RMSE of 0.18, and predict the 69% LAI variation with RMSE 0.01, and the results from airborne LiDAR based on regression agreewith the result from imaging spectrometry, and showed similar spatial patterns and ranges of values. Koetz [32] examined the feasibility of forest canopy structure characterization using LiDAR waveform model combined with the 3-D radiative transfer models. The forest biophysical parameters retrieved based on this method such as fractional cover, LAI, maximum tree height, and vertical crown extension are all good indicator for the horizontal and vertical forest canopy structure. High-density laser pulse returns of terrestrial LiDAR capture more details about the structure information of individual trees, hence, more and more researchers have begun to explore the advantages and potential power to retrieve biophysical parameters of forest ecosystems based on LiDAR [29–33]. Attention is not only placed on the leaf-on circumstance, but also the leaf-off and comparison between these two conditions [37, 38] in order to separate the points which could represent the photosynthetic parts of canopy. Leaves are usually a good indicator of environmental stresses, and LiDAR has been used to monitor forest stands and environmental changes through the indicator which LAI served as a key parameter [39].

##### Satellite

Due to the limited availability of satellite-based LiDAR systems specifically designed to study vegetation LAI discussion of research in this direction is not presented in this paper. Most of the study about retrieving LAI from LiDAR system still focuses on the airborne and terrestrial based LiDAR system, which serve as pioneer studies for satellite LiDAR system of the future. However, Radar system have been successfully used to retrieve LAI at the landscape level, for example, the radar backscatter of ERS-1 C-band VV polarization SAR data was used to acquire the LAI and soil moisture content in sugar beet field. The Leuwen and Clevers expression of the waster cloud model was fitted to determine the in situ relationship between radar backscatter and LAI. The results show considerable potential the operational application of ERS-1 SAR data in crop monitoring [116]. In addition, the EVNISAT Advanced Synthetic Aperture Radar (ASAR) vertical/horizontal (VV/HH) polarization ratio was correlated to the ground measured LAI for boreal forests with mean LAI estimation error 0.3 for Norway spruce (*Picea abies*(L.) Karst.) and 0.07 for Scots pine (*Pinus sylvestries* L.) [117].

### 3.3. Scaling Issues

Based on the discussion above, remote sensing techniques of different platforms such as aerial, ground-based, and space are used to rapidly retrieve LAI on the landscape scale, the sensors involves not only optical remote sensing, but also the active LiDAR system ranges from terrestrial, airborne, satellite based system. A wide range of spatial resolution images have been obtained by observing Earth using an array of remote sensing sensors. How to apply one algorithm developed for one scale to another scale successfully is a great issue due to the heterogeneity of land surface texture and non-linear estimation processes which will bias the biophysical parameter estimation based on the spatial resolution remote sensing images. Great amounts of work should be done to enhance the transportability of algorithms between different scales. In terms of LAI, the most important advantage of estimating LAI using remote sensing is that it remains consistent while the spatial scale changes. LAI has therefore been a meaningful biophysical parameter fit for multi-scale research ranges from plot to landscape level. It has been a convenient and ecologically-relevant variable for multi-scale studies that range from leaf to region [12]. From the spectral perspective, the mechanism of scaling from band to vegetation indices has been reported and discussed [118]. In order to map a biophysical parameter such as LAI to a landscape level based on remote sensing images, an appropriate scaling algorithm based on Landsat ETMdata has been developed based on sub-pixel information. The lumped calculation and distributed calculation were compared to find the bias that served as the coefficient to develop the scaling model [119]. In addition, a physically-based scaling with explicit spatial resolution dependent on radiative transfer formulation has been developed and applied to scaling LAI retrievals from NOAA-AVHRR data to other resolutions [120]. Regarding the scaling issue, heterogeneity is a key concept and the major source of error. There are two ways to scale transferring according to the direction of scaling: up-scaling and down-scaling, various LAI products are generated from remote sensing sensors with different spatial resolutions, when the LAI product with high resolution are transferred to low resolution by clumping scheme, the “up-scaling” term can be applicable to this method; for example, the LAI map from Landsat ETM with 30 × 30 m resolution can be up scaled to 1 km × 1 km images with multiple mosaic Landsat ETM images. Recently, a novel theory titled “spectral invariant theory” was developed [42], which is used to characterize the single scattering albedo of a volumetric element, which in essence can be tuned to account for differences in scaling and spectral sampling from different sensors to create long-term records of LAI from multiple sensors like AVHRR and MODIS. It also serves as a foundation to investigate the differences in spatial scaling and spectral band widths in introducing bias into LAI retrievals.

## 4. Conclusions

Direct and indirect terrestrial methods are the foundation and basis for obtaining accurate LAI estimates. The destructive sampling method for various forest tree species provides valuable data to validate results obtained from remote sensing platforms and algorithms. It is necessary to develop optical sensors that capture the light environment within the canopy which not only contains direct light from sunlight through the gaps of trees, but also diffused light and environmental light affections. Most of the optical instruments based on gap fraction theory infer effective LAI by recording the Photosynthetic Active Radiation (PAR), with these techniques it’s difficult to visualize the site environmental such as density and canopy height expect by utilizing hemispherical photography approach. Although a hemispherical photograph can permanently capture the light conditions at the moment that the picture is taken, it is difficult to control the exposure values and acquire every detail due to the resolution of which the digital camera is capable. Furthermore, the information obtained from digital hemispherical photographs is only two dimensional, thus, terrestrial LiDAR is an ideal instrument to complement photography by permanently recording the three-dimensional structural information. Some terrestrial LiDAR scanners are also capable of recording 360 degrees color images by setting up an appropriate exposure value (example: Leica ScanStation 2), however, the optical distortions in such imagery still needs to be reconciled. An instrument which not only records the Photosynthesis Photo Flux Density (PPFD), but also permanently captures the light conditions at the moment when the measurement is taken is ideal. Due to the non-random spatial distribution of leaves within a canopy, overlapping, and clumping effects, it is difficult to acquire true LAI despite removing partial effects based on the gap size analysis theory. Another problem of estimating LAI is the effect of branches and stems blocking the light. This blockage leads to the overestimation of LAI; thus far, the approach to remove this effect employs species-dependent empirical estimation of the fraction of non-photosynthetic portions of a tree. With the development of remote sensing technology, accurate, timely and dynamic acquisition of LAI at the landscape level requires us to develop new algorithms optimized for the characteristics of the remote sensors. Especially, with the emergence of LiDAR, we should synthesize the power to estimate the biophysical parameters of trees from forest stand level to landscape level, regional level, and even to global level by combing the spectral information of optical remote sensing and three-dimensional structure information from LiDAR. In such way we can provide more-accurate, timely and meaningful information for the development of ecological models and thus the dynamic monitoring of changes in an ecosystem.

## Figures and Tables

**Table 1.** Comparison of LAI definitions.

**Type**	**Definition**	**Application**	**Reference**
**Total Leaf Area Index (ToLAI)**	Total one-sided area of photosynthetic tissue per unit ground surface area.	Applicable to broad leaves.	[51,52]
**Projected Leaf Area Index (PLAI)**	The area of horizontal shadow that is cast beneath a horizontal leaf from a light at infinite distance directly above it.	Maximum area of leaves from the overhead orbital view – varies depending on the zenith angle of sensor.	[52–55]
**Silhouette Leaf Area Index (SLAI)**	The area of leaves inclined to the horizontal surface.	Investigates the radiation interception for different shapes of leaves.	[56]
**Effective Leaf Area Index (ELAI)**	One half of the total area of light intercepted by leaves per unit horizontal ground surface area – assume the foliage spatial distribution is random.	Precisely describes the radiation interception and radiation regime within and under canopy.	[57]
**True Leaf Area Index (TLAI)**	One half the total green leaf area per unit horizontal ground surface area.	Quantitatively characterizes radiation regime within and under canopy, and simulates leaf-controlled ecological process.	[58,59]
